# A3 INVESTIGATING MACROPHAGE-TISSUE CELL COMMUNICATION AXES IN THE GASTROINTESTINAL TRACT

**DOI:** 10.1093/jcag/gwad061.003

**Published:** 2024-02-14

**Authors:** L Ngai, A Mortha

**Affiliations:** Immunology, University of Toronto, Toronto, ON, Canada; Immunology, University of Toronto, Toronto, ON, Canada

## Abstract

**Background:**

The gastrointestinal tract boasts the highest concentration of macrophages (MPs) in the body, acting as vital immune sentinels that oversee tissue balance. Three distinct subsets of MPs, namely CCR2+, Tim4+, and CCR2,Tim4 double-negative (DN) MPs, populate distinct gut niches. Their functional specialization suggests unique interactions within their non-hematopoietic tissue microenvironments. Despite their crucial roles, the intricacies of these interactions remain elusive. One recently discovered interaction involves a communication axis between MPs and neurons within the muscularis layer of the gut. Here, neurons secrete Colony Stimulating Factor 1 (CSF1), a survival factor for MPs. These MPs reciprocate by producing Bone Morphogenetic Protein 2 (BMP2), assisting neurons in regulating gut motility.

**Aims:**

Based on the aforementioned interaction, we hypothesize that BMP2, derived from tissue-resident MPs, might influence functions of other tissue niches, subsequently prompting survival of MP via CSF1 production. Our aims are twofold: (1) Identify the role of BMP2 in gut macrophages; (2) Identify BMP2-responsive CSF1-producing tissue cells within the gut.

**Methods:**

To discern the roles of MP subsets, we developed a conditional knockout mouse model, targeting *Bmp2* expression in MPs. We validated successful *Bmp2* deletion(BMP2 KO) through quantitative polymerase chain reaction (qPCR) and an *in-situ* hybridization immunofluorescence assay (RNAscope). Subsequently, single cell RNA (scRNAseq) was performed, combining samples from both control and KO small intestines. This was done to further confirm BMP2 KO and to identify BMP2-responsive CSF1-producing cells. Flow cytometry was then used to characterize the intestinal MPs in control and KO mice.

**Results:**

Tim4+ MPs emerged as the primary source of BMP2. Comprehensive analysis using qPCR, RNAscope, and scRNAseq validated the complete KO of BMP2 across all macrophage subsets. The loss of MP-produced BMP2 led to a disruption in MP development. Notably, a marked reduction in DN and Tim4+ MPs was observed following deletion of *Bmp2* in MPs, emphasizing the critical role of BMP2-driven CSF1 in sustaining these subsets. CSF1 producing cells co-expressing the cognate BMP receptor in the gut, were predominantly non-hematopoietic tissue cells. Among these, distinct fibroblast populations emerged as a potent but not the sole source of CSF1.

**Conclusions:**

Our work underscores the involvement of multiple tissue cell populations, revealing an intricate network of interactions that extends beyond fibroblasts. This study unveils the multifaceted nature of tissue-MP interactions and their critical roles in maintaining gut homeostasis. Disturbances of these pathways may pave the way for disease by altering tissue homeostasis.

**Funding Agencies:**

NSERC

